# Gender inequality in the health workforce in the midst of achieving universal health coverage in Mexico

**DOI:** 10.1186/s12960-020-00481-z

**Published:** 2020-05-29

**Authors:** Julio César Montañez-Hernández, Jacqueline Elizabeth Alcalde-Rabanal, Gustavo Humberto Nigenda-López, Gladis Patricia Aristizábal-Hoyos, María Lorena Dino-Pou del Castillo

**Affiliations:** 1 National Institute of Public Health, Avenida Universidad 655, 62100 Col Santa María de Ahuacatitlán, CP Mexico; 2 National School of Nursing and Obstetrics, National Autonomous University of Mexico, Camino Viejo a Xochimilco y Viaducto Tlalpan, Huipulco, 14370 Mexico City, Mexico; 3 National Autonomous University of Mexico, Av. De Los Barrios 1, Hab. Los Reyes Ixtacala Barrio de los Árboles/Barrio de los Héroes, 54090 Tlalnepantla de Baz, State of Mexico Mexico; 4 Charité–Universitätsmedizin Berlin, corporate member of Freie Universität Berlin, Humboldt-Universität zu Berlin, and Berlin Institute of Health, Institut für Allgemeinmedizin, Charitéplatz 1, 10117 Berlin, Germany

**Keywords:** Gender inequality, Physicians, Nurses, Employment, Labor wastage, Desigualdad de género, médicos, enfermeras, mercado laboral, desperdicio laboral

## Abstract

**Background:**

The third Sustainable Development Goal aims to ensure healthy lives and to promote well-being for all at all ages. The health system plays a key role in achieving these goals and must have sufficient human resources in order to provide care to the population according to their needs and expectations.

**Methods:**

This paper explores the issues of unemployment, underemployment, and labor wastage in physicians and nurses in Mexico, all of which serve as barriers to achieving universal health coverage. We conducted a descriptive, observational, and longitudinal study to analyze the rates of employment, underemployment, unemployment, and labor wastage during the period 2005–2017 by gender. We used data from the National Occupation and Employment Survey. Calculating the average annual rates (AAR) for the period, we describe trends of the calculated rates. In addition, for 2017, we calculated health workforce densities for each of the 32 Mexican states and estimated the gaps with respect to the threshold of 4.45 health workers per 1000 inhabitants, as proposed in the Global Strategy on Human Resources for Health.

**Results:**

The AAR of employed female physicians was lower than men, and the AARs of qualitative underemployment, unemployment, and labor wastage for female physicians are higher than those of men. Female nurses, however, had a higher AAR in employment than male nurses and a lower AAR of qualitative underemployment and unemployment rates. Both female physicians and nurses showed a higher AAR in labor wastage rates than men. The density of health workers per 1000 inhabitants employed in the health sector was 4.20, and the estimated deficit of workers needed to match the threshold proposed in the Global Strategy is 70 161 workers distributed among the 16 states that do not reach the threshold.

**Conclusions:**

We provide evidence of the existence of gender gaps among physicians and nurses in the labor market with evident disadvantages for female physicians, particularly in labor wastage. In addition, our results suggest that the lack of physicians and nurses working in the health sector contributes to the inability to reach the health worker density threshold proposed by the Global Strategy.

## Resumen (Spanish)

**Antecedentes:**

El tercer Objetivo de Desarrollo Sostenible busca alcalzar vidas saludables y promover el bienestar para todos en todas las edades. Por lo cual, el sistema de salud es clave para lograr estos objetivos, y debe tener suficientes recursos humanos para brindar atención a la población de acuerdo con sus necesidades y expectativas.

**Métodos:**

Exploramos el desempleo, subempleo y desperdicio laboral en médicos y enfermeras en México como barreras para lograr la Cobertura Universal de Salud. Realizamos un estudio descriptivo, observacional y longitudinal para analizar las tasas de empleo, subempleo, desempleo y desperdicio laboral en el período 2005-2017, por género en ambas profesiones. Utilizamos la Encuesta Nacional de Ocupación y Empleo. Calculamos tasas anuales promedio (TAP) para el período para describir las tendencias. Además, para 2017 calculamos las densidades de la fuerza laboral para todos los 32 estados, y estimamos sus brechas con respecto al umbral de 4.45 propuesto en la Estrategia Global sobre Recursos Humanos para la Salud.

**Resultados:**

La TAP de las médicas empleadas era más bajo que el de los médicos, y la TAP del subempleo, el desempleo y el despilfarro laboral para las médicas eran más altas que en los hombres. Las enfermeras tienen una TAP más alta en el empleo que los enfermeros y una TAP más baja de subempleo y desempleo. Tanto las médicas como las enfermeras muestran unas TAP más altas en las tasas de desperdicio laboral. La densidad de trabajadores por cada 1,000 habitantes empleados en el sector de la salud fue de 4.2, y el déficit estimado para alcanzar el umbral propuesto en la Estrategia Global es de 70,161 trabajadores. Hipotéticamente, si todos los desempleados, subempleados y aquellos dedicados a las actividades del hogar estuvieran empleados en el sector de la salud, la densidad sería de 7.16.

**Conclusiones:**

Encontramos evidencia sobre la existencia de brechas de género entre médicos y enfermeras en el mercado laboral con desventajas evidentes para las médicas, y particularmente en el desperdicio laboral. Además, mostramos la falta de médicos y enfermeras trabajando en el sector de la salud para alcanzar el umbral propuesto en la Estrategia Global.

## Background

The third Sustainable Development Goal (SDG 3) aims to ensure healthy lives and promote well-being for all at all ages. To this end, all national governments have been called upon to achieve universal health coverage (UHC), which must include financial risk protection, access to basic quality health care services, and access to safe, effective, quality essential medicines. Consequently, health systems play a key role in achieving these ends, and therefore, they must have an adequate number of available and accessible human resources for health (HRH) to offer a wide variety of services to the population [1–3] and accelerate progress towards UHC [4, 5]. In addition, increases in HRH have been linked to a better quality of health services and increased development of solid and sustainable health systems [6].

The 2016 Human Resources for Health Report highlights the importance of aligning the health workforce with the population’s health needs, service coverage, and health outcomes [2, 7, 8]. Currently, most middle-income countries have a greater health workforce availability than a few years ago [3], which has been shown to positively impact health outcomes [6, 7]. Nevertheless, despite progress in HRH, there are still problems related to shortages of health workers, imbalances in geographical distribution, barriers to inter-professional collaboration, poor working conditions, unequal gender distribution, and limited availability of data regarding health workforce [9, 10].

Globally, we must address the HRH deficit, as well as inequalities in its distribution [11]. These problems with lack of medical personnel are widely recognized as the most insurmountable obstacles to improving health system performance and access to health services, especially in low- and middle-income countries (LMIC). Besides, the health workforce is changing its gender profile. While there is an increase of women in medicine, an increase in men has been reported in nursing [12]. These phenomena can, in part, be explained by changing gender roles in Western societies.

The World Health Organization (WHO) estimated that in order to achieve the SDGs by 2030 as planned, 18 million more health workers are needed in LMIC [6]. In the Global Strategy on Human Resources for Health (GSHRH), the WHO deemed that a density of 4.45 physicians, nurses, and midwives per 1000 inhabitants is the threshold required to achieve UHC [4].

In the Americas, about 70% of countries have enough physicians, nurses, and midwives to provide basic health services, but those countries still face challenges related to distribution, migration, and lack of training [13], especially in rural or high-marginalized areas [14].

In Mexico, in the 1990s, the unemployment rate of physicians was 12% while 8% had a job in a non-medical area [15]. Importantly, inactivity, unemployment, underemployment, and lower wages were concentrated in female physicians [16]. In 2008, 87% of physicians were working in the health sector and 10% had a non-medical job.

In Mexico, there is a great heterogeneity among nurses in terms of levels of training (i.e., technical, professional, and postgraduate) [17], which has contributed to the appearance of inequities in wages, as well as allocation and geographic distribution.

According to the Organisation for Economic Co-operation and Development (OCDE) [18], Mexico has a density above the threshold of 4.45 proposed in the GSHRH. However, we hypothesized that not all the 32 Mexican states reach the threshold due to unequal distribution throughout the country, and even if there was no unemployment, underemployment, and labor wastage in the health sector, some states of high marginalization would not reach the threshold. In addition, behind this hypothesis, we believe that although the number of professionals has increased over time, a possible cause of labor wastage in the country may be partly because women in both professions have fewer job opportunities and a large percentage of them are dedicated to household activities in a full-time basis.

## Methods

### Objective

The present study analyzes trends in employment, quantitative and qualitative underemployment, unemployment, and labor wastage rates for both physicians and nurses by gender between 2005 and 2017. Additionally, for 2017, we estimate the gap in the availability of HRH for each of the 32 Mexican states and compare it to the threshold proposed in the GSHRH, 4.45 health workers per 1000 inhabitants. For the second objective, we consider two scenarios: (a) calculating the densities only with the personnel employed in the health sector and (b) calculating the densities considering the entire available health workforce, including health personnel that are employed, unemployed, underemployed, and those dedicated to household activities on a full-time basis.

### Study design

We conducted a descriptive, observational, and longitudinal study to estimate the rates of employment, underemployment, unemployment, and labor wastage for both physicians and nurses from 2005 to 2017. For our estimates, we used data from the National Occupation and Employment Survey (ENOE, in Spanish). This survey uses a two-stage sampling, probabilistic design to enhance representativeness at the national and state levels. It is carried out with the objective of collecting information about the occupational characteristics of the Mexican population. The data is used to calculate and release official employment indicators. The units of analysis are households and the population aged 15+. Data is collected every 3 months using a longitudinal design, where, each quarter, 20% of households are replaced, so that each household remains in the sample for five quarters (INEGI) [19]. In order to describe trends, we analyze data from the third quarter (Trim-III) of each year from the period 2005–2017 (excluding each quarter of the remaining sample from the third quarter of the following year, except for 2017 where we take all households). The estimations for the size of the population are based on the projections of the National Population Council (CONAPO, in Spanish) [20] as well as the grouping of the states according to their level of marginalization (i.e., very high, high, medium, low, and very low) [21].

### Variables

We estimate the rates of employment, unemployment, underemployment, and labor wastage as well as the average annual rate (AAR) and the average annual growth rate (AAGR) from 2005 to 2017. Our definition of health workforce (HW) includes two occupational categories: physicians and nurses (including technicians) who have completed professional schooling. The following definitions are used to estimate the rates and constitute the employment pattern of the HW [15, 16, 22, 23]:

- Employed health workforce (E): physicians and nurses who work 20 h or more per week and perform health care or administrative functions in the health sector.
- Unemployed health workforce (U): physicians and nurses who were seeking work at the time of the survey because they were not linked to an economic activity.
- Quantitative underemployment (QnU): physicians and nurses whose work are underutilized because they work less than 20 h per week performing functions according to their profession or practice their profession as a secondary occupation.
- Qualitative underemployment (QlU): physicians and nurses who work but in a non-medical job.
- Household activities (H): physicians and nurses who exclusively perform household activities on a full-time basis.
- Potential health workforce (PHW): we included the following groups as potential workforce E, U, QnU, QlU, and H.

Employment, unemployment, underemployment, and labor wastage rates are calculated as follows:

- Rate of employment = $\frac{\mathrm{E}}{\mathrm{E}+\mathrm{QnU}+\mathrm{QlU}+\mathrm{U}}\times 1000$
- Rate of unemployment =$\frac{\mathrm{U}}{\mathrm{E}+\mathrm{QnU}+\mathrm{QlU}+\mathrm{U}}\times 1000$
- Rate of quantitative underemployment = $\frac{\mathrm{QnU}}{\mathrm{E}+\mathrm{QnU}+\mathrm{QlU}+\mathrm{U}}\times 1000$
- Rate of qualitative underemployment = $\frac{\mathrm{QlU}}{\mathrm{E}+\mathrm{QnU}+\mathrm{QlU}+\mathrm{U}}\times 1000$
- Rate of labor wastage = $\frac{\mathrm{QnU}+\mathrm{QlU}+\mathrm{U}+\mathrm{H}}{\mathrm{PHW}}$×1000

To summarize and describe the trends in rates over the period 2003–2017, the AAR and AAGR were estimated as follows:

- Average annual rate (AAR) = $\frac{\sum_{i=2005}^{2017}{\mathrm{rate}}_i}{13}$
- Average annual growth rate (AAGR) = $\frac{\sum_{i=2005}^{2016}\left({\mathrm{rate}}_{i+1}-{\mathrm{rate}}_i\right)}{12}$ (where *i* is the rate of E, U, QnU, QlU, and wastage)

Finally, in order to estimate the gap between the current number of health workers and the 4.45 per 1000 inhabitants threshold recommended in GSHRH, we estimate two measures for health workforce density:

- Employed health workforce density (EHWD) = $\frac{\mathrm{E}}{\mathrm{Total}\ \mathrm{population}}\times 1000$
- Potential health workforce density (PHWD) = $\frac{\mathrm{E}+\mathrm{QnU}+\mathrm{QlU}+\mathrm{U}+\mathrm{H}}{\mathrm{Total}\ \mathrm{population}}\times 1000$

### Statistical analysis

We performed the analysis on two levels. (1) At the national level, we estimated the rates of employment, quantitative and qualitative underemployment, unemployment, and labor wastage by gender and profession, and their AAR and AAGR from 2005 to 2017. (2) For 2017 and for each profession stratified by gender, we calculated the percentages and CI95% according to the employment pattern, age, schooling, and marital status; chi-square tests for nominal variables and Wilcoxon tests for ordinal variables were performed in order to assess the statistical differences. Further, at the state level, we estimated the percentages of employment and labor wastage for both physicians and nurses; states were grouped according to the level of marginalization, and we calculated the average percentage of each group.

Finally, considering the EHWD and PHWD for each state in Trim III-2017, we estimated the gap between each of these density indicators and the threshold of 4.45 per 1000 inhabitants as recommended in the GSHRH. The analyses were performed using STATA MP 13.0 accounting for the complex survey design, and statistical significance was set to the value *P* < 0.05.

## Results

During the period 2005–2017, the average annual rate (AAR) of employment was 792 per 1000 physicians and the average annual growth rate (AAGR) was 0.22%. Both quantitative and qualitative underemployment rates remained stable with no major changes (AAGR 0.89 and 1.81, respectively), and for every 1000 physicians, on average, 128 had a non-medical job (Table 1). The analysis of employment rates by gender reveals a lower AAR for women than for men (767 vs. 806). Likewise, the AAR and AAGR of qualitative underemployment and unemployment estimations for female physicians were higher than those for men.

**Table 1:** Physicians: rates of employment, quantitative and qualitative underemployment, and unemployment, by gender. Mexico 2005–2017

Rates × 1 000*	2005	2006	2007	2008	2009	2010	2011	2012	2013	2014	2015	2016	2017	AAR	AAGR
**All physicians**															
N*	**202 421**	**222 017**	**202 619**	**239 011**	**242 738**	**261 089**	**238 299**	**251 861**	**243 903**	**220 122**	**229 035**	**215 676**	**263 538**		
Employment	797	770	835	809	796	773	787	776	793	809	733	806	806	792	0.22
Quantitative underemployment	55	76	62	55	66	78	72	70	70	61	67	48	51	64	0.98
Qualitative underemployment	131	133	97	127	124	117	125	120	130	112	176	138	127	128	1.81
Unemployment	17	21	5	9	14	32	16	34	7	17	24	8	16	17	34.49
**Women**															
N*	**64 624**	**73 314**	**79 531**	**79 875**	**87 532**	**98 042**	**93 623**	**96 338**	**100 171**	**81 358**	**88 736**	**94 496**	**121 128**		
Employment	785	699	861	788	771	719	743	728	821	762	704	789	797	767	0.61
Quantitative underemployment	76	89	40	57	53	95	87	58	52	64	61	47	49	64	2.09
Qualitative underemployment	120	162	94	139	161	113	137	157	119	150	207	154	148	143	6.13
Unemployment	18	49	6	16	16	73	34	57	8	23	29	9	6	26	54.55
**Men**															
N*	**137 797**	**148 703**	**123 088**	**159 136**	**155 206**	**163 047**	**144 676**	**155 523**	**143 732**	**138 764**	**140 299**	**121 180**	**142 410**		
Employment	802	806	819	819	810	805	815	806	774	836	751	818	813	806	0.22
Quantitative underemployment	45	69	77	54	74	68	62	78	83	59	71	49	54	65	4.80
Qualitative underemployment	136	119	99	121	104	119	118	97	137	90	157	126	109	118	1.82
Unemployment	16	7	4	6	12	8	5	19	6	14	22	7	24	12	45.37

*AAR* average annual rate, *AAGR* average annual growth rate

*Professionals dedicated to household activities are not included in this table

Regarding nursing, the calculated AAR revealed that, for every 1000 nurses, there were 701 employed for 20 h or more per week, 43 were employed for fewer than 20 h, 226 held a non-medical job, and 30 were unemployed (Table 2). The analysis by gender showed that even though the employment rate of nurses was higher for women (706) than in men (654), the rate was decreasing for both genders. That said, the decrease is more rapid for men (AAGR = − 0.76%). The AAR and AAGR of qualitative underemployment and unemployment were lower for female nurses.

**Table 2:** Nurses: rates of employment, quantitative and qualitative underemployment, and unemployment rates, by gender. Mexico 2005–2017

Rates × 1 000	2005	2006	2007	2008	2009	2010	2011	2012	2013	2014	2015	2016	2017	AAR	AAGR
**All nurses**															
N*	**242 615**	**262 686**	**265 191**	**267 477**	**313 070**	**293 819**	**265 734**	**322 045**	**333 749**	**338 678**	**368 520**	**374 248**	**461 377**		
Employment	689	712	721	716	710	681	668	697	705	723	716	707	664	701	-0.26
Quantitative underemployment	51	37	25	48	51	59	46	40	49	34	42	33	46	43	4.16
Qualitative underemployment	232	233	225	210	208	209	261	227	226	219	212	222	248	226	0.95
Underemployment	28	18	29	26	30	51	25	36	20	23	29	38	41	30	10.76
**Women**															
N*	**229 379**	**243 848**	**244 208**	**243 485**	**288 128**	**264 242**	**243 441**	**282 406**	**296 571**	**290 456**	**320 630**	**320 293**	**390 397**		
Employment	684	721	737	716	717	681	664	703	723	729	721	712	674	706	-0.06
Quantitative underemployment	52	35	27	50	55	64	48	45	46	35	43	34	49	45	4.18
Qualitative underemployment	235	225	207	211	198	201	261	217	212	211	209	223	246	220	0.93
Underemployment	29	19	29	23	30	53	26	34	19	25	27	31	31	29	7.95
**Men**															
N*	**13 236**	**18 838**	**20 983**	**23 992**	**24 942**	**29 577**	**22 293**	**39 639**	**37 178**	**48 222**	**47 890**	**53 955**	**70 980**		
Employment	767	603	530	715	632	682	706	648	559	689	683	675	610	654	-0.76
Quantitative underemployment	42	65	9	23	3	10	21	4	77	29	38	26	29	29	170.96
Qualitative underemployment	183	332	436	202	327	274	260	300	333	270	237	219	262	280	8.92
Underemployment	8	0	25	60	39	35	13	47	32	12	42	80	99	38	54.17

*AAR* average annual rate, *AAGR* average annual growth rate

*Professionals dedicated to household activities are not included in this table

Table 3 shows that labor wastage rates in both physicians and nurses were greater for women than for men in almost every year of the 2005–2017 period. In 2005, women represented 35.4% and 95.8% of workforce among physicians and nurses, respectively, whereas in 2017, those percentages were 48.5% and 87.8%, respectively. Nevertheless, the AAR of labor wastage in both female physicians and female nurses is higher than men.

**Table 3:** Rates of labor wastage in physicians and nurses, by gender; Mexico

Rates × 1 000**	2005	2006	2007	2008	2009	2010	2011	2012	2013	2014	2015	2016	2017	AAR	AAGR
**Physicians (*N*)**	**216 073**	**235 672**	**219 424**	**255 268**	**262 094**	**284 615**	**264 306**	**266 915**	**264 245**	**241 550**	**246 442**	**234 733**	**292 035**		
Women (%)	**35.4**	**35.0**	**42.1**	**36.9**	**38.8**	**41.3**	**41.7**	**40.6**	**43.4**	**40.9**	**40.8**	**46.4**	**48.5**		
Men (%)	**64.6**	**65.0**	**57.9**	**63.1**	**61.2**	**58.7**	**58.3**	**59.4**	**56.6**	**59.1**	**59.2**	**53.6**	**51.5**		
Physicians (rate)	253	274	229	243	263	291	291	267	268	263	319	260	273	269	1.23
Women	336	379	258	332	336	400	369	352	282	372	379	315	318	341	1.31
Men	208	218	207	190	217	214	235	210	257	187	278	212	230	220	2.64
**Nurses (*N*)**	**313 245**	**337 709**	**335 117**	**351 807**	**393 197**	**368 618**	**359 431**	**388 177**	**428 357**	**436 853**	**455 892**	**482 463**	**591 801**		
Women (%)	**95.8**	**94.2**	**93.6**	**93.2**	**93.6**	**91.9**	**93.7**	**89.7**	**91.0**	**88.7**	**89.3**	**88.3**	**87.8**		
Men (%)	**4.2**	**5.8**	**6.4**	**6.8**	**6.4**	**8.1**	**6.3**	**10.3**	**9.0**	**11.3**	**10.7**	**11.7**	**12.2**		
Nurses (rate)	467	446	430	456	435	457	506	422	451	439	421	452	482	451	0.57
Women	477	448	426	468	439	469	520	429	450	454	432	464	493	459	0.63
Men	233	420	478	285	377	321	308	359	461	325	331	357	402	358	8.71

*AAR* average annual rate, *AAGR* average annual growth rate

**Includes employment, quantitative and qualitative underemployment, underemployment, and household activities

In 2017, in Mexico, there were 292 035 physicians and 591 801 nurses, of which 72.7% and 51.8% were employed 20 h or more in the health sector, respectively. By age, 16.8% of physicians and 27.7% of nurses were younger than 30 years of age, and 15.2% of physicians and 8.3% of nurses were 60 years and older. With regard to education, 25.7% of physicians were specialists or had postgraduate schooling, while 48.3% of nurses were technicians, and only 0.9% had a specialization (Table 4).

**Table 4:** Characteristics of physicians and nurses. Mexico, ENOE Trim III-2017

	Physicians			Nurses		
	Total	Women	Men	Total	Women	Men
*N*	**292 035**	**141 728**	**150 307**	**591 801**	**519 408**	**723 93**
*n*	**1 070**	**444**	**626**	**2 118**	**1 824**	**294**
%	**100.0**	**48.5**	**51.5**	**100.0**	**87.8**	**12.2**
**Employment pattern**						
Employment	72.7 [68.4, 76.6]	68.2 [60.6, 74.9]	77.0 [72.2, 81.2]*	51.8 [48.5, 55.0]	50.7 [47.3, 54.1]	59.8 [54.6, 64.8]*
Quantitative underemployment	4.6 [3.2, 6.6]	4.1 [2.6, 6.6]	5.1 [3.1, 8.3]	3.6 [2.5, 5.2]	3.7 [2.5, 5.5]	2.9 [1.5, 5.4]
Qualitative underemployment	11.5 [9.2, 14.2]	12.7 [9.3, 17.0]	10.3 [7.7, 13.6]	19.4 [16.7, 22.3]	18.5 [15.6, 21.7]	25.7 [20.7, 31.4]
Underemployment	1.4 [0.7, 2.8]	0.5 [0.2, 1.1]	2.3 [1.1, 4.8]	3.2 [2.0, 5.1]	2.3 [1.5, 3.5]	9.7 [8.2, 11.3]
Household activities	9.8 [7.5, 12.5]	14.5 [11.0, 18.9]	5.3 [3.3, 8.2]	22.0 [19.4, 24.9]	24.8 [21.9, 28.1]	2.0 [0.9, 4.3]
**Age group**						
20–29	16.8 [13.2, 21.2]	20.5 [14.2, 28.6]	13.4 [9.8, 18.1]*	27.7 [24.8, 30.9]	25.0 [22.2, 28.1]	47.2 [42.8, 51.6]*
30–39	24.7 [19.6, 30.7]	27.2 [19.1, 37.1]	22.4 [17.3, 28.3]	24.3 [21.4, 27.5]	24.1 [21.0, 27.6]	25.5 [19.4, 32.7]
40–49	21.8 [14.1, 32.2]	26.6 [13.0, 46.8]	17.4 [12.8, 23.2]	22.8 [19.9, 25.9]	23.2 [20.1, 26.5]	20.0 [12.9, 29.6]
50–59	21.4 [18.0, 25.2]	20.5 [15.6, 26.4]	22.3 [18.3, 26.9]	16.8 [14.4, 19.5]	18.2 [15.5, 21.2]	6.9 [5.4, 8.7]
60–69	12.7 [10.2, 15.7]	4.6 [2.9, 7.1]	20.3 [16.7, 24.5]	5.7 [4.0, 8.2]	6.5 [4.5, 9.3]	0.4 [0.3, 0.7]
70+	2.5 [1.6, 4.0]	0.7 [0.3, 1.8]	4.2 [2.5, 6.9]	2.6 [1.6, 4.3]	3.0 [1.8, 4.9]	
**Schooling**						
Technician				48.3 [45.1, 51.6]	49.0 [45.5, 52.5]	43.9 [38.9, 48.9]
Graduate	74.3 [65.0, 81.9]	74.0 [54.8, 86.9]	74.7 [69.1, 79.6]	50.8 [47.5, 54]	50.2 [46.7, 53.6]	55.0 [49.9, 60]
Specialty/postgraduate	25.7 [18.1, 35.0]	26.0 [13.1, 45.2]	25.3 [20.4, 30.9]	0.9 [0.5, 1.7]	0.9 [0.4, 1.7]	1.2 [0.4, 3.5]
**Marital status**						
No partner	36.3 [30.9, 42]	44.7 [34.4, 55.5]	28.3 [23.8, 33.3]*	43.0 [39.5, 46.6]	42.9 [39.1, 46.7]	44.1 [39.3, 48.9]
With partner	63.7 [58, 69.1]	55.3 [44.5, 65.6]	71.7 [66.7, 76.2]	57.0 [53.4, 60.5]	57.1 [53.3, 60.9]	55.9 [51.1, 60.7]
**Location**						
Rural	2.7 [2.4, 3.0]	2.0 [1.7, 2.5]	3.3 [3.1, 3.4]	6.5 [5.9, 7.1]	6.8 [6.2, 7.5]	3.8 [3.3, 4.3]
Urban	97.3 [97, 97.6]	98.0 [97.5, 98.3]	96.7 [96.6, 96.9]	93.5 [92.9, 94.1]	93.2 [92.5, 93.8]	96.2 [95.7, 96.7]

*P value < 0.05

For both physicians and nurses, women had lower percentages of employment (68.2 and 50.7%, respectively) compared to men (77.0 and 59.8%, respectively). A higher percentage of women were dedicated to household activities (14.5% of female physicians and 24.8% of female nurses compared to 5.3% male physicians and 2.0% of male nurses). Regarding age, 13.4% of male physicians were in the 20–29 age group, whereas 20.5% of female physicians were in that age bracket. For nurses, a lower percentage of female nurses were in the age bracket 20–25 (25.0%) compared to the percentage of men in that bracket (47.2%).

**Fig. 1:**
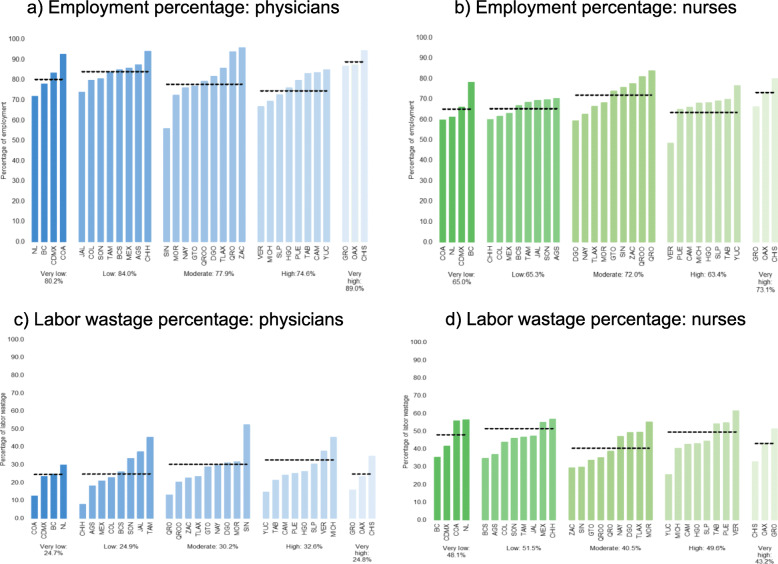
Rates of employment and labor wastage in physicians and nurses. ENOE TrimIII-2017

**Fig. 2:**
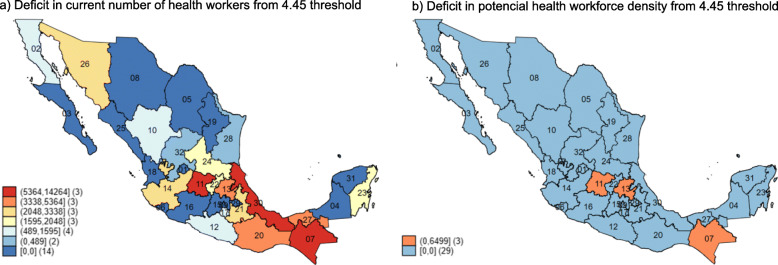
Deficit in the number of health workers from 4.45 threshold. ENOE TrimIII-2017

Figure 1 shows the heterogeneity of employment and labor wastage rates for physicians and nurses among the states grouped by level of marginalization. States with the highest marginalization had the highest employment average for both physicians and nurses (Fig. 1a, b). The highest physician labor wastage percentage was found in states with high and moderate marginalization (Fig. 2c). For nurses, the opposite was true, higher labor wastage percentages were found in the states categorized as low and very low categories (Fig. 2d).

In 2017, there were a total of 212 359 physicians (72.7%) and 306 491 nurses (51.8%) employed 20 h or more per week in the health sector in Mexico. Given that there is a population of 123.51 million, and that the EHWD per 1000 inhabitants was 4.2 (1.72 physicians and 2.48 nurses), we estimated a deficit of 70 161 workers to reach the threshold of 4.45 per 1000 as proposed by the GSHRH. At the state level, the estimated gap is heterogeneous. There were 18 states which did not reach the threshold, and the state deficits ranged from 87 workers to 14 264 workers (Fig. 2a). If we consider the potential health workforce density (PHWD), the density per 1000 inhabitants would reach 7.16 (Table 5) and only three states would still remain below the threshold (Fig. 2b). In this scenario, we calculated that a total of 8662 health workers would be required.

**Table 5:** Density of health workers by employment pattern. ENOE TrimIII-2017

Employment pattern	Number of health workers	Cumulative number of health workers	Density	Need	States under the 4.45 threshold
Employed (E)	518 850	518 850	4.2	70 161	18
Quantitative underemployment (QnU)	34 849	553 699	4.48	57 832	15
Qualitative underemployment (QlU)	148 031	701 730	5.68	23 174	8
Unemployment (U)	23 185	724 915	5.87	21 405	7
Household activities (H)	158 921	883 836	7.16	8 662	3

In 2017, the total population in Mexico in 2017 was 123 518 269

## Discussion

Our study showed that male physicians and female nurses have higher rates of employment compared to their female and male counterparts, respectively. Qualitative underemployment and unemployment are higher in female physicians and male nurses, and labor wastage is higher in both female physicians and female nurses. In this regard, a previous study in Mexico reported that qualitative underemployment among physicians was particularly higher in women in 2006, regardless of the type of academic qualifications, whereas, among nurses, qualitative underemployment was higher among men [22]. Ten years later, although the number of female physicians and male nurses has increased, gender discrepancies still exist, and underemployment and unemployment remain higher among female professionals. Moreover, some studies highlight wage differences in favor of male professionals [24, 25]. In addition, we found that quantitative underemployment is higher in female nurses. Some studies argue that women physicians are more likely to work part-time [26]. Both in medicine and nursing, common reasons for preferring part-time work include time to take care of children under 18 years old [27] or to continue with traditional household activities [28]. It is additionally highlighted that part-time physicians spend more time on teaching and research [26].

Regarding the feminization of physicians, a pattern is observed in most middle-income countries [29, 30] and is likewise becoming evident in Mexico. Between 2005 and 2017, the available workforce of female physicians in Mexico increased by 87% while available male physicians increased by only 3%. By 2017, in Mexico, almost half of the physicians employed in the health sector were women and around 75% of the health workforce (physicians and nurses) were women, with greater participation of women under 50 years than ever before. A similar trend has been observed in China, where about 51% of graduating physicians in 2005 were female, and by 2015, this percentage increased to 56% [31]. In general, the motivating factors for enrolling in medical schools are the scientific rigor of medicine and socioeconomic status and financial perspectives. For females, additional motivators include the social prestige of the profession, better opportunities to marry a professional [32], cultural preference for female physicians in conservative communities, and a desire to help poor people [33]. In response to this feminization of healthcare professions, some studies show positive and negative consequences. Researchers in Africa reported a trend that female physicians perform better standardized examinations [34], spending more time with their patients [35], writing fewer prescriptions, and referring cases more often [36]. However, some developing countries reported that female physicians work fewer hours and perform a lesser workload than their male counterparts [37, 38]. A systematic review showed a small negative impact of feminization on the availability of primary health care services in high-income countries [36].

About nursing in Mexico, we found that 12% of nurses were men in 2017, and the highest percentage of them were in the age category under 30 years old, indicating a recent trend in the growth of male nurses. A similar percentage is observed in the United States of America, where 13.6% of licensed nurses in 2015 were men [39]. The low numbers of males in nursing may be influenced by the social structure, given that this profession has been historically considered to be feminine [40]. Some authors argue that men reduce their social status when choosing the nursing profession, in contrast to women who raise their status [41]. In general, some studies explain gender discrimination in the health workforce due to the persistence of structural, social, and cultural factors that are perceived differently by stakeholders, physicians, and nurses [42].

We found a gap in training at the postgraduate level between physicians and nurses; while 25% of Mexican physicians had a postgraduate degree or specialty, only 1% of nurses had a specialty. This inequity in professional development is partly explained by the fact that the medical profession has specialty programs that are implemented under an academic program and is offered by public institutions with a monthly salary [43, 44]. In fact, we could identify only one relevant institutional initiative offering post-technical courses for nurses led by the Mexican Social Security Institute [17]. In summary, the results demonstrate important differences among physicians and nurses regarding gender and training level when they enter the labor market.

Finally, in terms of indicators of availability of health professionals, if we consider only those physicians and nurses employed in the health sector 20 h or more per week, Mexico has a density of 4.2 per 1000 inhabitants. Only 14 states achieved the threshold suggested in the GSHRH to reach UHC by 2030. If we consider also those who worked in the health sector less than 20 h per week, the density was 4.48 per 1000 inhabitants. The OECD reports a density of 5.33 for the same year but notes that interns and residents are included. In addition, the OECD report uses data from different sources, so double counting may occur as both physicians and nurses can work in the public and private sectors simultaneously [18]. When we consider qualitative underemployment and unemployment, the density was 5.87 and 7.16 per 1000 inhabitants  respectively,  when we included those dedicated to household activities on a full-time basis. Some studies argued that these negative indicators were due to the concentration of health workers in the areas of greatest development, which offer better opportunities for professional development and better wages [45]. Hence, it is necessary to develop strategies to incorporate into the labor market professionals that are part of labor wastage.

Although the GSHRH does not mention what proportions of physicians and nurses would be adequate, systematic review informs that collaboration between physicians and nurses can have a positive impact on patient outcomes and a variety of pathologies [46]. Further, they conclude that properly trained nurses are capable of as high-quality care as primary care physicians and achieve equally good results in patients [47].

Our study has some limitations. Our estimation of rates of employment does not consider physicians and nurses who participate in teaching and research activities, so quantitative underemployment may be overestimated. Furthermore, it is not possible to distinguish between those who choose to work less than 20 h and those who have to do so because of market conditions. In addition, the analysis at the state level only considers one trimester, and it is not possible to identify labor mobility that can affect rates and state densities over time. Perhaps, in the future, if we choose another quarter or year, such as 2007 or 2014, when employment was at its highest rates, the national density of HRH would reach the threshold of 4.45 per 1000 for those who worked 20 h or more. Subsequent analyses of the time series could help explain possible seasonal cycles in the behavior of rates.

## Conclusion

We provide evidence on the existence of gender gaps among physicians and nurses in the labor markets. The rates of employment were higher in men, and rates of unemployment and labor wastage were higher in women. This indicator pointed the disadvantages of the health labor market in Mexico for women, and this phenomenon particularly affects nurses where most of them are female. In general, if the health workers employed 20 h or more per week are considered, the gap to reach the WHO threshold is small; however, this gap decreases as the labor wastage enters the labor market. Therefore, policies on human resources for health should be oriented towards the incorporation of labor wastage in the labor market and the achievement of gender equity in relation to job responsibilities, promotion, retention, and remuneration [48].

## Data Availability

The datasets analyzed during the current study are available in the National Institute of Statistics and Geography repository [https://www.inegi.org.mx/programas/enoe/15ymas/]. All data generated during this study are included in this published article and its supplementary material files.

## Abbreviations

AAR: Average annual rate
AAGR: Average annual growth rate
CONAPO: National Population Council
ENOE: National Survey of Occupation and Employment
EHWD: Employed health workforce density
GSHRH: Global Strategy on Human Resources for Health
HRH: Human Resources for Health
HW: Health workforce
INEGI: National Institute of Statistics and Geography
LMIC: Low- and middle-income countries
OECD: Organisation for Economic Co-operation and Development
PHWD: Potential health workforce density
PHW: Potential health workforce
SDG: Sustainable Developing Goals
UHC: Universal health coverage
WHO: World Health Organization
