# Correction to: Gender inequality in the health workforce in the midst of achieving universal health coverage in Mexico

**DOI:** 10.1186/s12960-020-00486-8

**Published:** 2020-06-12

**Authors:** Julio César Montañez-Hernández, Jacqueline Elizabeth Alcalde-Rabanal, Gustavo Humberto Nigenda-López, Gladis Patricia Aristizábal-Hoyos, Lorena Dini

**Affiliations:** 1 National Institute of Public Health, Avenida Universidad 655, 62100 Col Santa María de Ahuacatitlán, CP Mexico; 2 National School of Nursing and Obstetrics, National Autonomous University of Mexico, Camino Viejo a Xochimilco y Viaducto Tlalpan, Huipulco, 14370 Mexico City, Mexico; 3 National Autonomous University of Mexico, Av. De Los Barrios 1, Hab. Los Reyes Ixtacala Barrio de los Árboles/Barrio de los Héroes, 54090 Tlalnepantla de Baz, State of Mexico Mexico; 4 Charité–Universitätsmedizin Berlin, corporate member of Freie Universität Berlin, Humboldt-Universität zu Berlin, and Berlin Institute of Health, Institut für Allgemeinmedizin, Charitéplatz 1, 10117 Berlin, Germany

**Correction to: Human Resources for Health (2020) 18:40**

**https://doi.org/10.1186/s12960-020-00481-z**

Following publication of the original article [1], the authors identified an error in the author name of Lorena Dini.

1. Incorrect name:

María Lorena Dino-Pou del Castillo

2. Correct name:

Lorena Dini

The author group has been updated above and the original article [1] has been corrected.
